# Toxicity and Anti-Proliferative Properties of *Anisomeles indica* Ethanol Extract on Cervical Cancer HeLa Cells and Zebrafish Embryos

**DOI:** 10.3390/life11030257

**Published:** 2021-03-20

**Authors:** Nguyen T. Bich-Loan, Kieu Trung Kien, Nguyen Lai Thanh, Nguyen T. Kim-Thanh, Nguyen Quang Huy, Pham The-Hai, Marc Muller, Amandine Nachtergael, Pierre Duez, Nguyen Dinh Thang

**Affiliations:** 1 Faculty of Biology, VNU University of Science, Vietnam National University, Hanoi 100000, Vietnam; nguyenthibichloan.iph@gmail.com (N.T.B.-L.); kieukien1@gmail.com (K.T.K.); nguyenlaithanh@hus.edu.vn (N.L.T.); kimthanh_bio@yahoo.com (N.T.K.-T.); huy_nq@hus.edu.vn (N.Q.H.); hai.phamthe@gmail.com (P.T.-H.); 2 Unit of Therapeutic Chemistry and Pharmacognosy, University of Mons (UMONS), 7000 Mons, Belgium; Amandine.NACHTERGAEL@umons.ac.be (A.N.); Pierre.DUEZ@umons.ac.be (P.D.); 3 Laboratory for Organogenesis and Regeneration, GIGA-R, Department Life Sciences, University of Liege, 4000 Liege, Belgium; m.muller@uliege.be

**Keywords:** medicinal plant, *Anisomeles indica*, HeLa cell, zebrafish, apoptosis, cell cycle arrest, gene expression, colony formation

## Abstract

In this study, we showed that crude extract of *Anisomeles indica* (AI-EtE) expressed its toxicity to HeLa cells with an IC50 dose of 38.8 µg/mL and to zebrafish embryos with malformations, lethality and hatching inhibition at 72-hpf at doses higher than 75 µg/mL. More interestingly, flow cytometry revealed that AI-EtE significantly promoted the number of cells entering apoptotic. Accordingly, the transcript levels of *BAX*, *CASPASE-8*, and *CASPASE-3* in the cells treated with AI-EtE at IC50 dose were 1.55-, 1.62-, and 2.45-fold higher than those in the control cells, respectively. Moreover, treatment with AI-EtE caused cell cycle arrest at the G1 phase in a p53-independent manner. Particularly, percentages of AI-EtE-treated cells in G1, S, G2/M were, respectively 85%, 6.7% and 6.4%; while percentages of control cells in G1, S, G2/M were 64%, 15% and 19%, respectively. Consistent with cell cycle arrest, the expressions of *CDKN1A* and *CDNK2A* in AI-EtE-treated cells were up-regulated 1.9- and 1.64-fold, respectively. Significantly, treatment with AI-EtE also decreased anchorage-independent growth of HeLa cells. In conclusion, we suggest that *Anisomeles indica* can be considered as a medicinal plant with a possible use against cervical cancer cells; however, the used dose should be carefully monitored, especially when applying to pregnant women.

## 1. Introduction

A recent survey showed that 64% of new drugs discovered between 1981 and 2010 were natural products or at least inspired by them [1,2]. Several products/medicines used for treatments of different cancers had been extracted from medicinal plants such as paclitaxel from the bark of western yew tree *Taxus brevifolia* [3], capsaicin from the hot pepper of the genus *Capsicum* [4], and resveratrol from the skin of grapes [5]. Thus, the importance of putting new efforts into development and use of traditional medications has been highlighted in recent recommendations [6,7].

Vietnam represents a rich source of endemic and potentially novel natural medicines. Many plant extracts have been used as traditional medicines for thousands of years. However, so far, the use of traditional medicines has been relying mostly on empirical observations. Toxicities, as well as the biological and pharmaceutical properties of the medicinal plant extracts have not been thoroughly investigated yet. Thus, administrations of these natural products for a long period of time may also lead to more or less adverse effects on human health.

Cervical cancer is the fourth most common cancer in women. Recent statistics revealed that cervical cancer caused about 7.5% of all female cancer deaths; of those deaths, approximately 90% occurred in the developing countries [8,9]. In Vietnam, it was similarly estimated that cervical cancer was the fourth most common cancer in Vietnamese women with more than 5000 new cases and about 3000 deaths in 2018 [8].

Cell cycle arrest and apoptosis are the two main reasons for cell death, therefore they are always considered as endpoints for screening of drugs for cancer treatments. Apoptosis is initiated by intrinsic and/or extrinsic pathways, which finally promote the activities of caspases to damage the cellular DNA and cause cell death [10,11,12]. On the other hand, the cell cycle is regulated by Cyclin-Dependent Kinases (CDKs), which are activated at specific points during progression of the cell cycle. Arresting the cell cycle at any phase of G1, S, and G2/M of the cell cycle inhibits cell division and may ultimately cause cell death [13,14,15,16,17,18].

*Anisomeles indica* is one of the medicinal plants displaying different biological activities including antioxidant [19,20], antibacterial [20,21] and anticancer [22,23]. Previous studies demonstrated that the products originating from *Anisomeles indica* could induce the death of lung cancer cells [22,23], oral cancer cells [24], and liver cancer cells [10] by inducing cell cycle arrest and activating the apoptosis pathway. *Anisomeles indica* was also able to inhibit the migration and invasion activities of breast cancer cells [25]. However, the molecular mechanisms underlying these actions have not been thoroughly investigated. In addition, so far, no study addressed the anticancer activity of *Anisomeles indica* on cervical cancer cells nor its putative toxicity on human embryos. An effective and popular in vivo experimental model for drug screening and, in particular, embryotoxicity, is the zebrafish [26,27]. Therefore, in this study, we examined the biological and pharmaceutical properties, focusing on cell-targeted toxicity (cultured cells), organism-targeted toxicity (zebrafish embryos) and anticancer activity on cervical cancer in vitro, of the crude extracts of the medicinal plant *Anisomeles indica*, which has been used as traditional medicine in the North-West of Vietnam for a long time.

## 2. Materials and Methods

### 2.1. Plant Material

Medicinal plants (Table 1) were selected based on the recipes of traditional medicines and collected from the North-Western mountainous areas of Vietnam in the dry season in 2019. They were identified, coded (with Vietnamese and Scientific names) and placed at the Museum of Biology, Faculty of Biology, VNU University of Science, Vietnam National University, Hanoi, Vietnam.

### 2.2. Preparation of Plant Extracts

**Figure 1:**
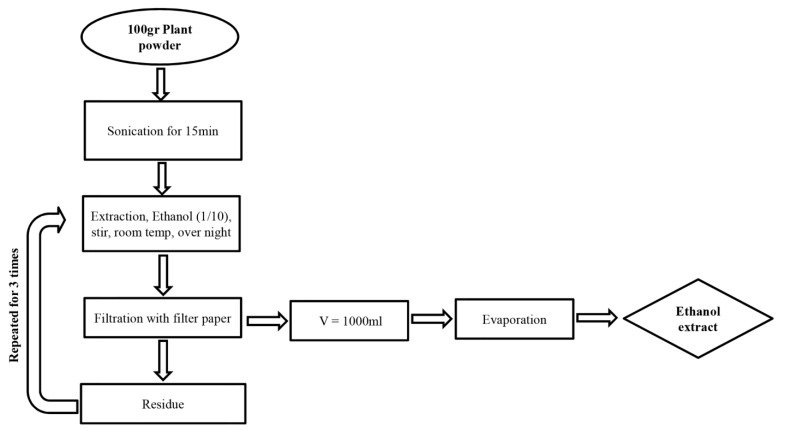
Extraction scheme for plant powder.

Fresh medicinal plants were cleaned and washed thoroughly with water and re-washed with distilled water. Washed fresh leaves were shade-dried, powdered mechanically, and sieved by using a mesh. For preparation of organic solvent extracts, 100 g of powdered material was mixed with 1000 mL of ethanol, sonicated for 15 min in a sonication bath (with set-up conditions of 35 °C; 40 kHz frequency and 150W ultrasonic power) [28], before extraction for overnight with stirring at room temperature. The resulting extract was removed, and the procedure was repeated two additional times on the solid residue. All extracts were filtered, pooled, and the solvent removed under reduced pressure at 40 ± 5 °C using a rotary flash evaporator. The scheme of the extraction procedure is presented in Figure 1. The obtained extract was dissolved in DMSO for further analysis and exposure on cells and zebrafish eggs.

### 2.3. Cell Culture

Cervical cancer HeLa (ATCC: CCL-2) and breast cancer MCF-7 (ATCC: CRL-3435) cell lines were provided by Health Science Research Resources Bank, Japan and cultured in DMEM and RPMI-1640 (respectively), supplemented with 10% Fetal Bovine Serum (FBS) and 1% Penicillin/Streptomycin at 37 °C in 5% CO_2_.

### 2.4. Embryo Zebrafish Toxicity Test

Adult zebrafish wild type strain AB (*Danio rerio*) (ZIRC, Eugene, OR) [26] were maintained in the zebrafish facility of the Animal Laboratory. The fish were cultured in glass rectangular pools measuring 40 cm (wide) × 50 cm (long) × 30 cm (high). Several pools of adult fish were bred individually for each assay. After sorting, embryos from pools with high fertility (facil were mixed and used for subsequent experiments. Experiments were validated only when the survival rate of the controls was ≥ eri at 4 days post fertilization (dpf). Fish were reared in a Techniplast recirculating system under 14:10-h light/dark photocycle. Before use, eggs were screened and sorted under a stereoscope to remove the unfertilized and/or abnormal ones. Healthy embryos that showed normal cleavage were distributed into 6-well plates at 25 embryos/well for the embryotoxicity tests.

### 2.5. Cellular Toxicity MTT Assay

MTT (Sigma Chemical Co., St. Louis, MO, USA) assays were conducted as previously described [29] with some modifications. Briefly, cells were seeded into 96-well plates. The MTT stock solution of 5 mg/mL was prepared in PBS (pH 7.2) and filtered through 2 µm pore-size membrane. At the end of the treatment period with extracts (12, 24 or 48 h), 20 µL of MTT solution were added to each well and incubated for 4 h at 37 °C. Then, 100 µL of solubilizing buffer (prepared by dissolving of 10% sodium dodecyl sulfate SDS in 0.01 N HCl) were added to each well and incubated overnight. After that, the 96-well plate was read using an ELISA reader at 570 nm for absorbance density values. Viable cells produce a dark blue formazan product, whereas no such staining was formed in dead cells. The percentage of viable cells was calculated.

### 2.6. Annexin V Apoptosis Assay

The annexin V apoptosis assay was performed as described by Schutte [30]. It is based on the detection of phosphatidylserine, present on the extracellular side of the cell membrane only in apoptotic cells, by FITC-conjugated annexin V. The cells were seeded at 30 to 40% confluence in 6-cm plates. After overnight incubation, medium was aspirated and replaced with medium with or without extract. Treatment with paclitaxel was used as a positive control for apoptosis induction. After 36 h, medium was collected. The cells were washed with PBS and the collected cells were resuspended in annexin binding buffer (Thermo Fisher Scientific, Singapore) at 1 × 10^6^ cells/mL. Cells were stained with propidium iodide (Invitrogen, Thermo Fisher Scientific, Singapore) to label dead or late apoptotic cells, and annexin V-FITC according to the manufacturer’s protocol and assayed by flow cytometry on a FACSCanto II (BD Biosciences). The percentage of apoptotic cells was measured as the percentage of annexin V-positive cells.

### 2.7. Cell Cycle Analysis

Cells were stained with propidium iodide (PI) (BD Biosciences) to measure the DNA content using a flow cytometer (FACs CANTO system) [31]. Briefly, cells collected by trypsinizing were washed twice with cold PBS buffer (pH 7.4) and two volumes of cold 100% ethanol were added. After ethanol fixation, the cells were centrifuged at 400 g for 5 min and washed once in the PBS buffer. Cells were then re-suspended at 10^6^ cells/mL. Fifty μg/mL of RNase A (Sigma Chemical Co., St. Louis, MO, USA) was added to each sample before incubating at 37 °C for 30 min. After incubation, 20 μg of PI was added to each tube and kept for at least 30 min to provide the nuclear signal for fluorescence-activated cell sorting. After staining the cells with PI, the tubes were transferred to ice, protected from light, and used for cell cycle analysis on the flow cytometer.

### 2.8. Real-Time PCR Analysis

Total RNA was isolated from cells or zebrafish larvae using the RNA Isolation Kit (Thermo Fisher Scientific, Singapore), according to the protocol of the Kit. Then cDNA was synthesized from total RNA by reverse transcription, according to the protocol of the cDNA Synthesis Kit (Thermo Scientific, USA). Real-time quantitative RT-PCR with SYBR green was performed using power SYBR1 green PCR master mix in a LightCycler@96 Instrument (Roche Diagnostics GmbH, Mannheim, Germany). The transcript expression levels of *BAX*, *CASPASE-3*, *CASPASE-8*, *p53*, *CDKN1A* (*p21*) and *CDKN2A* (*p16*) were measured by quantitative RT-PCR (real-time PCR) were standardized relative to the transcript level of TBP. Real-time PCR was carried out using 10 μL of power SYBR1 green PCR master mix containing 900 nM forward primer and 900 nM reverse primer in a final volume of 20 μL. The sequences of the primers are presented in the Supplementary Materials, Table S1. After running, the amplification curve, melting peak, and melting curve were analyzed to confirm the reliability of the results and to ensure that there are no noise signals which may come from amplifications of primer dimer and/or other side reactions.

### 2.9. Colony Formation Assay

A colony formation assay was performed to assess the development of tumor in vitro, according to the previous report [32]. Briefly, after pre-treating with the AI-EtE or drug for 24 h, 5 × 10^4^ cells were mixed with 2 mL of 0.36% soft agar in RPMI medium, poured onto slightly solid 0.72% hard agar in RPMI medium pre-seeded on the 6-well plate, and then cultured for 3 weeks. Medium was added on the surfaces of the agar layer every two days. Colonies were examined under microscope every three days. At day 21, colonies exceeding 50 µm in diameter were counted and presented as an activity of anchorage-independent growth.

### 2.10. Statistical Analysis

Statistical analysis in this study was performed as previously described [33]. Results from three independent experiments in each group were statistically analyzed by a Student’s *t*-test. The SPSS (version 18) software package (SPSS Japan Inc., Tokyo, Japan) was used for statistical analyses, and the significance level was set to *p* < 0.05.

## 3. Results

### 3.1. Anisomeles Indica Extract Displayed the Highest Cytotoxicity on Cervical Cancer HeLa Cells

**Figure 2:**
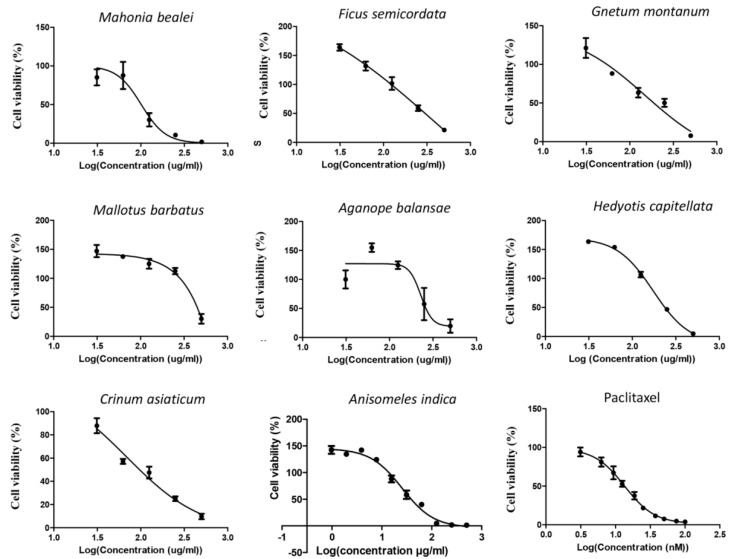
HeLa cells were treated with the indicated extracts at various concentrations (1, 5, 10, 50, 100, 250, 500 and 1000 µg/mL) and MTT survival tests were performed. The toxicity curves of the ethanol extracts of *Mahonia bealei, Ficus semicordata, Gnetum montanum, Crinum asiaticum, Mallotus barbatus, Aganope balansae, Hedyotis capitellata, Anisomeles indica* and Paclitaxel are presented. The cell viability is shown as % relative to untreated control. Cell viability curves were drawn using GraphPad Prism software.

**Figure 3:**
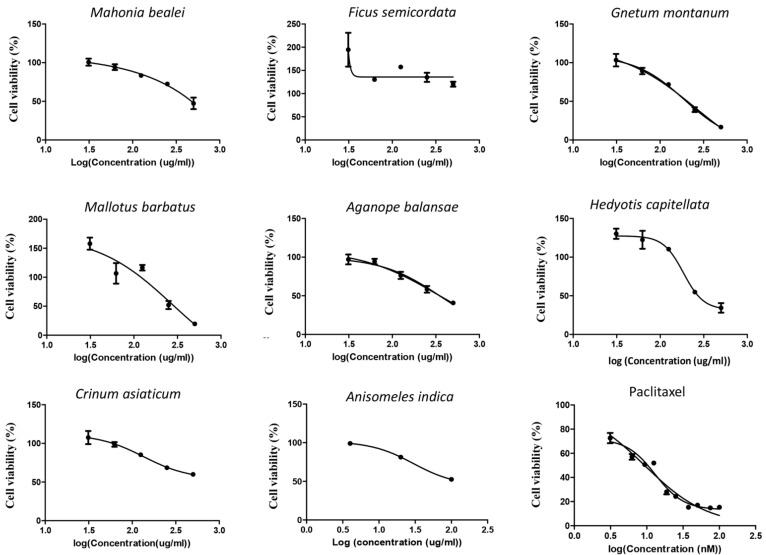
The MCF-7 cells were treated with the extracts at various concentrations of 1, 5, 10, 50, 100, 250, 500 and 1000 µg/mL. The toxicity curves of the ethanol extracts of *Mahonia bealei*, *Ficus semicordata*, *Gnetum montanum*, *Crinum asiaticum*, *Mallotus barbatus*, *Aganope balansae*, *Hedyotis capitellata*, *Anisomeles indica* and Paclitaxel were presented. The cell viability is shown as % relative to untreated control. The curves were drawn by using GraphPad prism software.

Ethanol extracts of eight medicinal plants, including Anisomeles indica, Mahonia bealei, Ficus semicordata, Gnetum montanum, Crinum asiaticum, Mallotus barbatus, Aganope balansae, and Hedyotis capitellata were used for screening of their toxicity on cervical cancer HeLa and breast cancer MCF-7 cells. Treatment with paclitaxel was used as a positive toxicity control. The observed toxicity curves in HeLa and MCF-7 cells are shown in Figure 2 and Figure 3, respectively.

Based on these dose-response curves, the 50% inhibitory concentration (IC50) and correlation coefficient (R^2^) values for all extracts were determined as summarized in Table 2. Among these eight plant extracts, the ethanol extract of *Anisomeles indica* (AI-EtE) displayed the highest toxic activity on HeLa cells, with an IC50 value of 38.8 µg/mL, followed by the ethanol extract of *Crinum asiaticum* (IC50 = 69.5 µg/mL) and *Mahonia bealei* (IC50 = 101 µg/mL) (Figure 2, Table 2). A very low toxicity was observed for all the extracts on the MCF-7 cells, again the AI-EtE was the most toxic with IC50 of 133 µg/mL (Figure 3, Table 2). Paclitaxel (taxol), a drug approved by FDA for cancer treatment and used as a positive control, had its IC50 values at 13.5 ng/mL and 15 ng/mL on HeLa and MCF-7 cells, respectively (Figure 2 and Figure 3, Table 2). Generally, a substance with IC50 value in the range of 0–50 μg/mL is normally considered as a toxic agent; therefore the AI-EtE was used for further experiments.

### 3.2. Toxicity of Anisomeles Indica Ethanol Extract on Zebrafish Embryos

**Figure 4:**
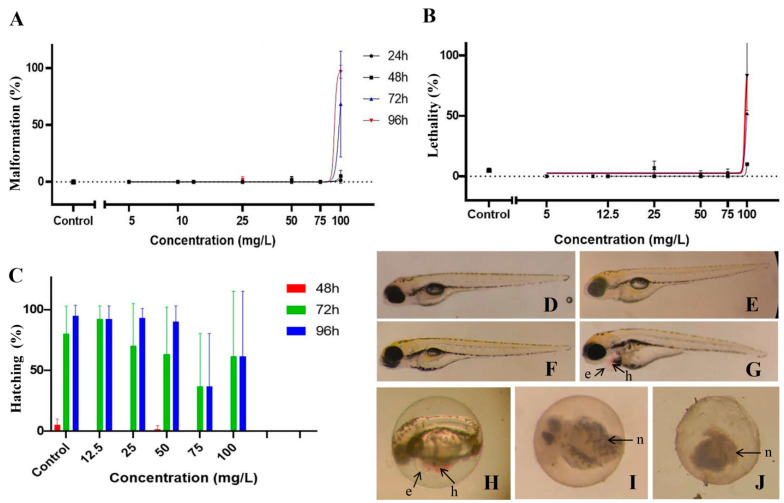
Effect of AI-EtE on development and survival of zebrafish embryos at different time points. The effects of AI-EtE at various doses (0, 5, 10, 12.5, 25, 50, 75, 100, 200 and 400 mg/L) on defects during development (**A**), lethality (**B**), and hatching percentage (**C**) of zebrafish embryos at 24, 48, 72 and 92 hpf, respectively, are presented as dose-response curves. Representative images of 96 hpf zebrafish larvae presenting various developmental defects upon AI-EtE exposure at different doses, (**D**) 0 mg/L (negative control), (**E**) 25 mg/L, (**F**) 50 mg/L, (**G**) 75 mg/L, (**H**) 100 mg/L, (**I**) 200 mg/L, and (**J**) 400 mg/L. Illustrated defects on larvae include yolk-sac oedema (**e**), haemovascular defect (**h**), and necrosis (**n**) as indicated with arrows.

Experiments were conducted to evaluate the effect of AI-EtE on the development of zebrafish embryos at different stages. AI-EtE at various doses of 0, 5, 10, 12.5, 25, 50, 75, 100, 150, 200 and 400 (mg/L) were applied. At each time point (24, 48, 72, and 96 h post-fertilization (hpf)), the presence of developmental defects and lethality of the zebrafish embryos (larvae) were assessed (Figure 4A,B). The specific endpoint of hatching was also assessed at 48, 72 and 96 hpf (Figure 4C). At concentrations up to 75 mg/L, AI-EtE had almost no effect on the development of defects or death of zebrafish larvae with malformation of 5%, lethality of 2.5%, and hatching efficiency of 70% (Figure 4A–C). However, at 100 mg/L, a sharp increase in developmental malformations (Figure 4A) with haemovascular defects and heart/yolk sac oedema on 75% larvae at 72 hpf and 90% larvae at 92 hpf (Figure 4G,H). Similarly, at doses above 100mg/L, a sharp increase in embryo and larva death was observed at each observation time point (Figure 4B). At 150 mg/L, 200 mg/L and 400 mg/L, AI-EtE was very toxic to zebrafish embryos because of the death of all embryos at very early time (Figure 4H–J). In addition, we monitored the hatching percentage of the embryos, which normally takes place between 48 and 72 hpf, as illustrated in the control fish (Figure 4C). Treatment with AI-EtE had a negative effect on the hatching ability in a dose-dependence manner. Specifically, at 75 mg/L, AI-EtE strongly decreased the hatching percentage of zebrafish embryos to around 30% at both 72 hpf and 96 hpf (Figure 4C).

### 3.3. The Anisomeles Indica Ethanol Extract Induced HeLa Cell Apoptosis

**Figure 5:**
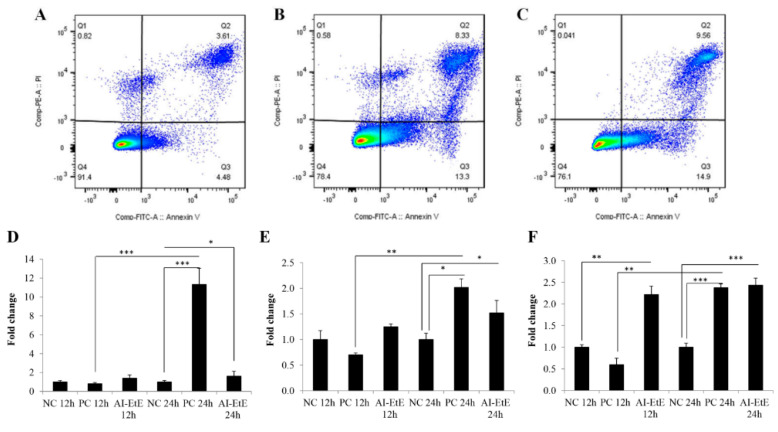
HeLa cells were treated with DMSO (negative control), AI-EtE at IC50 dose of 38.8 mg/L or paclitaxel (positive control) at IC50 dose of 13.5 ng/mL for 24 h before collecting, fixing, staining with propidium iodide (PI)/annexin V-FITC and subjecting to flow cytometry system to measure of apoptosis. Representative data sets of apoptotic pattern of HeLa cells upon DMSO treatment (**A**), AI-EtE treatment (**B**), and paclitaxel treatment (**C**) are presented. Transcript levels of *BAX* (**D**), *CASPASE-8* (**E**), *CASPASE-3* (**F**) in HeLa cells treated with AI-EtE and paclitaxel for 12 h and 24 h. *, ** and ***, significant differences with *p* values < 0.05, <0.01 and <0.001, respectively. TBP was used as internal control gene. NC: negative control, PC: positive control (paclitaxel).

We also tested the effect of drug/extract on cell apoptosis by treating HeLa cells with AI-EtE and paclitaxel for 24 h at the IC50 doses. Flow cytometry of the cells after staining with propidium iodide (PI) and annexin V-FITC revealed the percentage of cells in necrosis (Q1), late apoptosis (Q2), early apoptosis (Q3), and living cells (Q4) (Figure 5A–C). We observed that the percentage cells entering apoptosis (Q2) was 3.0-fold higher in AI-EtE-treated cells compared to control cells (Figure 5A,B), while paclitaxel increased the percentage of apoptotic cells up to 3.3-fold (Figure 5A,C).

Cell apoptosis may be initiated by intrinsic and/or extrinsic signaling pathways. Therefore, we investigated the transcript levels of genes that play important roles in apoptosis pathways in HeLa cells by real-time PCR analysis. The obtained results demonstrate that the transcript levels of *BAX*, *CASPASE-8*, and *CASPASE-3* were up-regulated in the AI-EtE-treated HeLa cells in a dose-dependent manner (Figure 5D–F). After 12 h and 24 h treatment with AI-EtE, the expression levels of *BAX*, *CASPASE-8*, and *CASPASE-3* genes were up-regulated 1.34-, 1.23- and 2.33-fold (at 12 h) and 1.55-, 1.62- and 2.45-fold (at 24 h), respectively (Figure 5D–F). It was also revealed that the effects of AI-EtE and paclitaxel on apoptosis-related gene expressions had similar patterns; however, paclitaxel was more effective on the intrinsic pathway than on the extrinsic pathway, as shown by the massive increase of the *BAX* transcript level (Figure 5A).

### 3.4. The Anisomeles Indica Ethanol Extract Induced HeLa Cell Cycle Arrest at the G1 Phase

**Figure 6:**
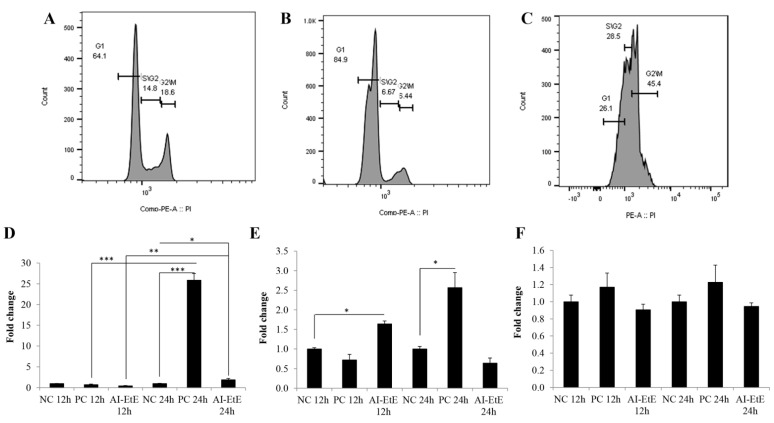
HeLa cells were treated with DMSO (negative control), AI-EtE at IC50 dose of 38.8 mg/L or paclitaxel (positive control) at IC50 dose of 13.5 ng/mL for 24 h before collecting, fixing, staining with propidium iodide and subjecting to flow cytometry system to measure of cellular DNA content. Representative data sets of cell cycle pattern of HeLa cells upon DMSO treatment (**A**), AI-EtE treatment (**B**), and paclitaxel treatment (**C**) were presented. Transcript levels of *CDKN1A* (**D**), *CDKN2A* (**E**) and *p53* (**F**) in HeLa cells treated with AI-EtE and paclitaxel for 12 h and 24 h. *, ** and ***, significant differences with *p* values <0.05, <0.01 and <0.001, respectively. TBP was used as internal control gene. NC: negative control, PC: positive control (paclitaxel).

HeLa cells were treated with AI-EtE or paclitaxel at the IC50 doses for 24 h. The cells were stained with propidium iodide that directly binds to the DNA in the nucleus of ethanol-fixed cells. DNA contents were measured by flow cytometry to determine the proportion of cells in G1, S, and G2/M phases. The DNA content histograms reveal that in the negative control batch (Figure 6A), the percentages of HeLa cells distributed in the G1, S, and G2/M phases were around 64%, 15% and 18%, respectively; while, in the AI-EtE-treated batch (Figure 6B), the percentages of HeLa cells in the G1, S, and G2/M phases were 85%, 7% and 6.5%, respectively. These results indicate that AI-EtE significantly induced cell cycle arrest in the G1 phase and consequently decreased the number of cells entering the S and G2/M phases. In contrast, paclitaxel arrested the cell cycle at the G2/M phase, as indicated by the percentages of cells distributed in the G1, S, and G2/M phases of 26%, 29% and 45%, respectively (Figure 6C).

Finally, to define the molecular events causing the cell cycle arrest, the transcript levels of genes coding for proteins regulating the cell cycle, such as *CDKN1A (p21^CIP1^)*, *CDKN2A (p16^INK4A^)* and *p53* were investigated. Interestingly, treatment with either AI-EtE or paclitaxel did not affect the transcript levels of *p53* in HeLa cells (Figure 6F). However, consistent with G1-phase retardation, the transcript levels of *CDKN1A* and *CDKN2A* in the AI-EtE-treated cells were up-regulated 1.64-fold at 24 h and 1.90-fold at 12 h, compared with those in the control cells, respectively (Figure 6D,E). On the other hand, paclitaxel strongly increased the expression levels of both *CDKN1A* and *CDKN2A* genes at the time of 24 h of 25.9- and 2.57-folds, respectively (Figure 6D,E).

### 3.5. AI-EtE Decreased Anchorage-Independent Growth Ability of HeLa Cells

**Figure 7:**
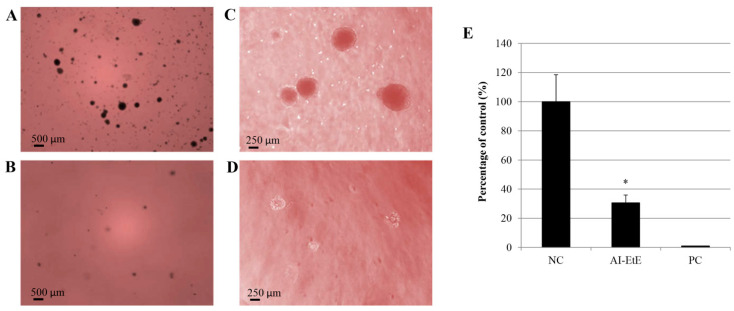
Anchorage-independent growth of HeLa cells. Colonies formed on the soft agar in case of control cells (**A**,**C**) and AI-EtE-treated cells (**B**,**D**) at magnifications of 2X (**A**,**B**) and 5X (**C**,**D**). The differences in colony formation ability on soft agar of HeLa cells are presented in a graph (**E**). *, significant differences with *p* values < 0.05.

We next performed a colony formation assay to investigate anchorage-independent growth of HeLa cells. The obtained results demonstrated that exposure to AI-EtE at IC50 dose of 38.8 mg/mL decreased colony formation ability as well as reduced the size of colonies of cells in soft agar (Figure 7B,D) compared with control cells (Figure 7A,C). Particularly, number of colonies in case of control cells was 3.5-fold higher than that in AI-EtE-treated cells (Figure 7E). Meanwhile, treatment with paclitaxel at the IC50 dose of 13.5 ng/mL totally inhibited colony formation of cells (Figure 7E).

## 4. Discussion

Nowadays, natural products or medicines originating from natural sources are attracting the interest of scientists all over the world. Thus, in a recent report, WHO insisted on the importance to develop and use traditional medications [6]. However, the effects of traditional medicines on human health are not always supported by hard scientific evidence and the molecular mechanisms they rely on are rarely assessed. In this study, we screened the toxicity and the anticancer activity of crude extracts from eight medicinal plants, which have been used as traditional medicines in North-Western Vietnam, on the cervical cancer HeLa cell line. We found that an ethanolic extract from *Anisomeles indica* (AI-EtE) was the only one presenting a high toxicity on HeLa cells, with an IC50 value of 38.8 mg/L (Figure 2).

Previous studies indicated that extracts and products isolated from *Anisomeles indica* (AI) had many biological effects in vitro. Particularly, aqueous extract of AI expressed anticancer activity via inhibition of metastatic activity of breast cancer cells [25], different AI extracts had antimicrobial and antioxidant properties [20,21,22,23]. Further, chemical compositions of AI had also been investigated, and ovatodiolide, a macrocyclic diterpenoid compound, was one of the most important chemicals that possessed various biological activities [22,23,24,25,34,35]. Ovatodiolide expressed its anticancer properties by initiation of apoptosis via a ROS-dependent ATM/ATR signaling pathways, induction cell cycle arrest at G2/M phase of breast, lung and oral squamous carcinoma cancer cells [22,24,25,35]. However, so far, the effects of AI extracts on cervical cancer cells have not been examined yet, thus, in this study, we focused our investigation on the anticancer effects of this AI-EtE on HeLa cancer cells.

Apoptosis induces distinct biochemical and morphological changes in the cell and can be triggered by internal and/or external factors. The intrinsic pathway is initiated by activation of the pro-apoptotic (*BAX*) leading to the release of cytochrome C from the mitochondria [10,11,12]. The extrinsic pathway, triggered mainly by extracellular signals, activates *CASPASE-8*. Both pathways will finally lead to activation of the execution caspase *CASPASE-3*, which in turn degrades the cellular DNA and causes cell death [10,11,12]. Our results show that AI-EtE could induce cell apoptosis up to 2.7-fold (Figure 5A) via activating both *BAX* (Figure 6A) and *CASPASE-8*, as well as increase the expression of the down-stream *CASPASE-3*. Thus, AI-EtE activates both the intrinsic and extrinsic apoptotic pathways. Similarly, paclitaxel triggered apoptosis by both intrinsic and extrinsic pathways; however, its effect was much stronger on the intrinsic pathway as shown by the more intense increase in expression of *BAX* as compared to *CASPASE-8*) (Figure 5C and Figure 6A,B). In addition, it had been revealed that AI-EtE could induce the transcript expressions of CASPASE-3 at the early time (12 h); on the other hand, although paclitaxel had almost no effect on transcription levels of BAX, CASPASE-8 and CASPASE-3 at 12 h, it strongly increased the expressions of these genes at 24 h, especially in the case of BAX (Figure 6A–C). These results might also support for the fact that AI-EtE induced cell arrest at the early time (G1 phase) while paclitaxel promoted cell arrest at the late time (G2/M phase).

Although, previous studies found that ovatodiolide isolated from n-hexane extract of *Anisomeles indica* caused the cell arrest at the G2/M phase of the cell cycle in cases of lung cancer cells [22], breast cancer [25,35] and oral cancer cells [24]. In this study, we found that AI-EtE might promote the HeLa cell arrest at the G1 phase rather than at the G2/M phase of the cell cycle (Figure 5B). This means that there may be other compounds in the ethanol extract of *Anisomeles indica*, which can act on cell cycle arrest at G1 or it may be because of the different behavior of HeLa cells compared with other cancer cells. We further investigated the expression levels of genes encoding proteins involved in regulation of the cell cycle, including p53, CDKN1A (p21^CIP1^), and CDKN2A (p16^INK^). p53 is generally involved in cell cycle arrest induced by DNA damage [13], and can also stimulate the transcription of *CDKN1A* and BAX [14,15]. However, in this study, the transcript expression of p53 in HeLa cells was not affected by treatment with ether AI-EtE or paclitaxel (Figure 6F), suggesting that neither of them induced DNA damage. However, both AI-EtE and paclitaxel strongly increased the transcript levels of *CDKN1A,* which mainly responds for G1/S phase and *CDKN2A,* which normally has dominant expression at the G2/M phase (Figure 6D,E). Previously, p21 has been reported as a key molecule, which plays an important role in regulation of the critical G1 to S phase transition of the cell cycle, senescence and apoptosis, and to be positively regulated by p16 [16]. Moreover, previous studies demonstrated that p21 could be up-regulated and resulted in cell cycle arrest at G1 phase in a p53-independent manner [17,18,36]. In addition, it had been also revealed that paclitaxel could induce the cell apoptosis with a p53-independent way [37]. Normally, the expressions of p53 and p16 are in a motive balance, a decrease of p53 leading to an increase of p16 [38]. Thus, in this study, the obtained results suggested that the AI-EtE contributed in regulation of cell cycle via activating the expressions of cyclin-dependent kinase inhibitors p21 and p16. Moreover, CDK also contribute in regulating the cyclin protein levels rise and fall during the cell cycle [39]. Different cyclins are required at different phases of the cell cycle [40,41,42], particularly, mitosis of cell is regulated by cyclins in complex with CDK1 [43,44]. So, consistent with previous report, this study suggested that the induction of p21 and p16 might have inactivated CDK and resulted in cell cycle arrest. Along with invasion activity, anchorage-independent growth is a hallmark of cancer cells. It represents the proliferative ability of cancer cells in the absence of adhesion to extracellular matrix proteins and correlates closely with tumorigenesis [32,45,46]. To investigate the anchorage-independent growth, colony formation (tumor formation in vitro) assay had been carried out. The ability in inhibiting the colony formation of cancer cells is one of the most important effects of drug for cancer treatment, especially for cancers with solid tumors [32,45,46]. In this study, we found that AI-EtE was not only significantly decreased in the number of colony formation of HeLa cells on soft agar but also strongly reduced the size of colonies. Although there had been reports that products extracted from AI were able to decrease invasion activity of several cancer cells [25,35], there is no report about the effect of AI originated products on anchorage-independent growth of cancer cells.

Zebrafish is an effective in vivo model for whole animal screening for developmental defects because of its advantageous properties such as high throughput, high similarity of the genome with the human one, rapid development, transparency of the embryos making them easy to observe and manage [26,27]. In this study, the effects of AI-EtE on zebrafish embryos at 24, 48, 72 and 96 hpf were examined. We found that AI-EtE at the concentrations below 80 mg/L had almost no effect on zebrafish embryo development and survival. Only a decrease in hatching ability was observed at a dose of 75 mg/L. In contrast, however, at higher concentrations starting at 100 mg/L, AI-EtE strongly induced developmental defects and death of the larvae. Further investigations about the effect of AI-EtE in vivo are needed.

Based on the chemical properties, ovatodiolide is mainly distributed into n-hexane, however, the extraction protocol presented in previous study [47] was described with following steps: firstly, ethanol was used for crude extraction, and after ethanol evaporation, the pellet was collected for next extraction steps in different solvents such as n-hexane, and ethyl acetate to collect several fractions [47]. This means that the ethanol crude extract surely contains an amount of ovatodiolide. Moreover, other phytochemicals such as phenolics and flavonoids are easily dissolved in ethanol/methanol [20,28,47,48,49,50], and therefore they certainly present in the ethanol extract. In fact, previous studies found that ovatodiolide (isolated from Anisomeles indica) induced cell cycle at G2/M arrest in lung cancer cells [22] and squamous carcinoma cancer cells [24]; while, in this study, we revealed that the Anisomeles indica ethanol extract induced HeLa cell arrest at G1. It implies that there is not only ovatodiolide but also other phytochemicals could effect on HeLa cancer cell and/or zebrafish embryos. Although the obtained results from this study demonstrated that the AI-EtE has possible use in traditional medicine against cervical cancer, in the future studies, chemical compositions of AI-EtE should be addressed by using HPLC and/or HPTLC methods [51,52] to identify the phytochemicals that are able to be used as fingerprints for quality control of the AI-EtE.

## 5. Conclusions

Taken together, we suggest that *Anisomeles indica* can be considered as a medicinal plant and that *Anisomeles indica* ethanol extract is able to use as a traditional medicine against cervical cancer cells; however, the real impact of this extract on cancer remain to be evaluated through epidemiological data and pharmacokinetic studies coupled with clinical trials. Moreover, a cautious approach is advised with the used dose, especially when applying for pregnant women, because of its ability in causing hatching inhibition, the development of defects and death of zebrafish larvae.

## Figures and Tables

**Table 1:** Medicinal plants used for cytotoxicity screening.

	Vietnamese Name	Scientific Name	Code in Museum	Place for Collection	Part Used
1	Phòng phong thảo	*Anisomeles indica*	HNU 024777	Bát xát, Lào Cai	Dry stem leaves
2	Hoàng liên ôro lá dày	*Mahonia bealei*	HNU 024779	Bát xát, Lào Cai	Dry stem
3	Đa lá lệch	*Ficus semicordata*	HNU 024780	Vị Xuyên, Hà Giang	Dry leaves
4	Gắm núi	*Gnetum montanum*	HNU 024781	Vị Xuyên, Hà Giang	Dry stem
5	Náng hoa trắng	*Crinum asiaticum*	HNU 024783	Cẩm Phả, Quảng Ninh	Dry leaves
6	Bùm bụp	*Mallotus barbatus*	HNU 024784	Lạc Sơn, Hòa Bình	Dry root
7	Mạn mân	*Aganope balansae*	HNU 024785	Bắc Quang, Hà Giang	Dry stem
8	Dạ cẩm	*Hedyotis capitellata*	HNU 024786	Bắc Quang, Hà Giang	Fresh leaves

**Table 2:** IC50 values of different ethanol extracts on HeLa and MCF-7 cells.

No	Sample	HeLa		MCF-7	
		IC _50_ (µg/mL)	R^2^	IC _50_ (µg/mL)	R^2^
1	*Anisomeles indica*	38.8	0.92	133	0.99
2	*Mahonia bealei*	101	0.84	254	0.90
3	*Ficus semicordata*	358	0.96	188	0.86
4	*Gnetum montanum*	150	0.93	257	0.96
5	*Crinum asiaticum*	69.5	0.93	215	0,95
6	*Mallotus barbatus*	498	0.98	276	0.84
7	*Aganope balansae*	228	0.71	310	0.91
8	*Hedyotis capitellata*	173	0.99	184	0.94
	Paclitaxel	13.5 (ng/mL)	0.96	15 (ng/mL)	0.98

## Data Availability

Publicly available datasets were analyzed in this study.

## Supplementary Materials

The following are available online at https://www.mdpi.com/2075-1729/11/3/257/s1. Table S1. Primer sequences of genes used in the study.
