# Supplementary material for: Toxicity and Anti-Proliferative Properties of Anisomeles indica Ethanol Extract on Cervical Cancer HeLa Cells and Zebrafish Embryos
Source: Life (Basel). 2021 Mar 20;11(3):257. doi: 10.3390/life11030257 (PMC8003830; doi:10.3390/life11030257)
Supplement: Supplementary file 1 [file life-11-00257-s001.pdf]

Supplemental table 1: Primer sequences of genes used in the study

Gene	Primer	Sequence
P53	Forward	**5-CCTCAGCATCTTATCCGAGTGG-3**
	Reverse	**5-TGGATGGTGGTACAGTCAGAGC-3**
CASPASE-3	Forward	**5-GGAAGCGAATCAATGGACTCTGG-3**
	Reverse	**5-GCATCGACATCTGTACCAGACC-3**
CASPASE-8	Forward	**5-AGAAGAGGGTCATCCTGGGAGA-3**
	Reverse	**5-TCAGGACTTCCTTCAAGGCTGC-3**
BAX	Forward	**5-TGACGGCAACTTCAACTGGG-3**
	Reverse	**5-AGCACTCCCGCCACAAAGA-3**
CDKN1A ($p21^{Cip1}$)	Forward	**5-**AAGTCAGTTCCTTGTGGAGC**-3**
	Reverse	**5-**ATTAGCGCATCACAGTCGCG**-3**
CDKN2A ($p16^{INK4A}$)	Forward	**5- CAACGCACCGAATAGTTACG-3**
	Reverse	**5- TGCCCATCATCATGACCTGG-3**
TBP	Forward	**5-CACGAACCACGGCACTGATT-3**
	Reverse	**5-TTTTCTTGCTGCCAGTCTGGAC-3**
